# Cultural adaptation and content validity evidence of the Nutritional Literacy Scale Brazilian version

**DOI:** 10.1590/0034-7167-2021-0657

**Published:** 2022-07-18

**Authors:** Christiane Pineda Zanella, Helena Alves de Carvalho Sampaio, José Wellington Oliveira de Lima, Thereza Maria Magalhães Moreira

**Affiliations:** I Universidade Estadual do Ceará. Fortaleza, Ceará, Brazil

**Keywords:** Nutritional Sciences, Food and Nutrition Education, Health Literacy, Validation Studies, Psychometrics., Ciencias de la Nutrición, Educación Alimentaria y Nutricional, Alfabetización en Salud, Estudios de Validación, Psicometría., Ciências da Nutrição, Educação Alimentar e Nutricional, Letramento em Saúde, Estudos de Validação, Psicometria.

## Abstract

**Objective:**

To investigate the evidence of content validity and reliability of the Brazilian version of the Nutritional Literacy Scale (NLS) after the cultural adaptation process.

**Methods:**

Psychometric study of 1,197 users of the National Health Service (Brazilian SUS). The NLS was culturally adapted to Brazilian Portuguese and six items of the original scale were modified to improve its understanding, giving rise to the Brazilian version of the scale named NLS-BR. The analysis of evidence of content validity of the NLS-BR was performed using the Item Response Theory (IRT).

**Results:**

The final version of the NLS-BR had 23 items and proved to be adequate to assess nutritional literacy in adults assisted by the Brazilian SUS.

**Final considerations:**

The NLS-BR proved to be of adequate understanding and demonstrated evidence of content validity and reliability for users of the Brazilian SUS.

## RESUMO

**Objetivo:**

Investigar evidências de validade de conteúdo e confiabilidade da versão brasileira da Nutritional Literacy Scale (NLS) após processo de adaptação cultural.

**Métodos:**

Estudo psicométrico com 1197 usuários do Sistema Único de Saúde (SUS). A NLS foi adaptada culturalmente para a língua portuguesa do Brasil e seis itens da escala original foram modificados para melhorar seu entendimento, originando a versão brasileira da escala, denominada NLS-BR. A análise das evidências de validade de conteúdo da NLS-BR se deu via Teoria de Resposta ao Item (TRI).

**Resultados:**

A versão final da NLS-BR ficou com 23 itens e se mostrou adequada para avaliar letramento nutricional em adultos brasileiros atendidos pelo SUS.

**Considerações finais:**

A NLS-BR mostrou-se de entendimento adequado, podendo ser considerada com evidências de validade de conteúdo e dotada de confiabilidade para usuários do SUS.

## RESUMEN

**Objetivo:**

Investigar las evidencias de la validez de contenido y confiabilidad de la versión brasileña de la Nutritional Literacy Scale (NLS) después del proceso de adaptación cultural.

**Métodos:**

Estudio psicométrico con 1197 usuarios del Servicio Nacional de Salud (SUS brasileño). La NLS fue adaptada culturalmente al portugués brasileño y seis ítems de la escala original fueron modificados para mejorar su comprensión, dando origen a la versión brasileña de la escala, denominada NLS-BR. El análisis de evidencia de validez de contenido del NLS-BR se realizó utilizando la Teoría de Respuesta al Ítem (TRI).

**Resultados:**

La versión final de la escala NLS-BR tenía 23 ítems y se mostró adecuada para evaluar la educación nutricional en adultos atendidos en el SUS brasileño.

**Consideraciones finales:**

La escala NLS-BR demostró ser de comprensión adecuada y tener evidencias de validez de contenido y confiabilidad para los usuarios del SUS brasileño.

## INTRODUCTION

Food inadequacy, one of the biggest public health problems nowadays, partially reflects the lack of knowledge of topics related to nutrition and is a relevant cause of development of chronic conditions. Health Literacy (HL) has been a theoretical framework to support the confrontation of this problem worldwide.

According to Sorensen et al.^(1)^, HL involves people’s knowledge, motivation and skills to access, understand, evaluate and apply health information in order to make judgments and everyday decisions about health care, disease prevention and health promotion to maintain or improve quality of life. When specifically focused on nutrition, it is called Nutritional Literacy (NL). More recently, the term Food Literacy (FL) has been adopted as a construct that encompasses nutritional literacy. In this perspective, NL refers only to the skills to understand nutritional information, while FL comprises: 1) the ability to read, understand and judge the quality of nutritional information (which corresponds to NL); 2) to seek and exchange knowledge about food and nutrition; 3) to buy and prepare food; 4) critically reflect on factors involved in personal food choices; and 5) to understand the impact of these choices on society^(2)^. Only the term nutritional literacy will be adopted in the present study, since it is used both in the scale adapted here and in more traditional texts on the subject.

The study of HL has grown worldwide since the end of the 20^th^ century, impacting on the availability of instruments for its measurement. Some instruments validated in Brazil are examples, such as: Test of Functional Health Literacy in Adults-TOFHLA^(3)^, validated by Maragno et al.^(4)^; Health Literacy Test-TLS; Newest Vital Sign-NVS^(5)^, validated as NVS-BR^(6)^; Short Assessment of Health Literacy for Spanish-Speaking Adults-SAHLSA^(7)^, validated as SAHLPA^(8)^; and more recently, the Health Literacy Questionnaire-HLQ^(9)^, validated as HLQ-Br^(10)^. In relation to NL, as comparatively, there are fewer instruments, it is difficult to measure this construct, and the existing instruments use the term NL because the conceptual change under analysis is recent^(2)^.

Although limited, as it only allows analyzing the understanding of nutritional information, the NVS can also be used to assess NL. One of the most cited instruments in the literature is the Nutrition Literacy Assessment Instrument - Nlit^(11-12)^. This instrument is used to assess the understanding of nutritional information, and has domains on understanding food groups and portions, and skills as a consumer. It is a long instrument (66 items) culturally adapted in Brazil, but not yet published in any scientific journal^(13)^. The pilot test performed by the Brazilian author showed an application time of 50.57 (15.45) minutes, which she considered a limiting factor for its use in practice^(13)^.

The interest in the Nutritional Literacy Scale arose within this perspective. This instrument was developed in 2007^(14)^, has 28 items that assess the understanding of nutritional information and its relationship with health and disease. According to its author, the scale has acceptable psychometric characteristics^(14)^. It was also validated in Spanish for Latin adults^(15)^, for the Greek population^(16)^, and described by the authors as appropriate and useful.

## OBJECTIVE

To investigate the evidence of content validity and reliability of the Brazilian version of the Nutritional Literacy Scale (NLS) after the cultural adaptation process.

## METHODS

### Ethical aspects

The subjects signed an informed consent form. The development of the study continued after approval from the Research Ethics Committee of the Universidade Estadual do Ceará (UECE). Resolutions nº 510/16 and 466/12 of the National Health Council, which provide for the Regulatory Guidelines and Norms for Research involving Human Beings, were respected.

### Design, period and place of study

Analytical study conducted in 2015 with users of the National Health Service (Brazilian SUS) in primary care units of Regional Departments I, II, III, IV, V and VI of the Municipal Health Department of Fortaleza. Participants responded to the Brazilian version (NLS-BR) of the Nutritional Literacy Scale after its translation and cultural adaptation to Brazil.

Worldwide, the NLS is the most traditional instrument for measuring NL. It was developed by Diamond^(14)^, who validated his 28-item scale to measure NL in the United States. In the present study, the NLS was obtained free of charge, after sending a request to the author via e-mail (james.diamond@jefferson.edu). The Brazilian version (NLS-BR) started with the translation and adaptation of the original scale to Brazil, following the five steps by Beaton et al.^(17-18)^: 1) Initial translation; 2) Synthesis of the translation; 3) Back-translation to the original language; 4) Evaluation by a committee of judges; and 5) Pre-test of the final version.

The initial translation was performed by two Brazilians with mastery of the English language, duly registered, independent certified translators, originating versions T1 and T2. They did not know the objectives of the scale, which was sent to them by email.

The committee of judges (three nutritionists, a language professional and the two translators involved in the development of T1 and T2) analyzed the clarity and coherence of the content of the two versions in Portuguese in comparison with the original version in order to minimize the possibilities of typical translation errors, such as omission or addition of words and expressions that could change the meaning of items. In addition, the semantic (word meaning), idiomatic (expressions difficult to translate), cultural (items reflecting experiences of each culture) and conceptual (term/expression evaluating the same aspect in both cultures) equivalence of the instrument were analyzed in modified Delphi (two meetings). Regarding instruments in the health area, the committee of judges must have at least one professional with methodological knowledge, a language professional and a health professional, and translators^(18)^. The three nutritionists were professors of undergraduate nutrition courses and one of them was a professor of the Postgraduate Program in Public Health. All three nutritionists developed research on the topic of health and nutrition literacy financed by national funding agencies (Ministry of Health-MS; National Council for Scientific and Technological Development-CNPq; and Ceará Foundation for the Support of Scientific and Technological Development-FUNCAP).

Based on the analysis of versions T1 and T2, a single version was developed (T12) and forwarded to the back-translation step. This step was carried out separately by two independent professionals, native speakers of English (BT1 and BT2) and certified translators. The T12 version was sent by email. The committee of judges that also included the two subjects responsible for the back-translation, evaluated BT1 and BT2 versions to define the final version of the instrument, named NLS-BR. The content aspect was covered without use of a specific instrument, according to the adopted reference that does not foresee the use of an instrument at this step. The recommendations of judges were all accepted before the pre-test.

As recommended in the literature^(17-18)^, the pre-test of the NLS-BR version was performed with 30 users of the Brazilian SUS for assessment of their understanding of items, among other things. Items understood by 85% of the 30 users were considered as appropriate. Each respondent was asked to report their understanding of each question and responses were noted to assess understanding. As four out of the 28 items of the first version of the NLS-BR (expert recommendations on lipid reduction, fiber function, correct food storage and organic feeding) were not understood, they were changed and again presented to the 30 users, reaching a satisfactory understanding. Note that this sample of 30 people was not included in the final sample of the study.

Next, the scale was applied to 1,197 adult users of the Brazilian SUS. Twenty Primary Health Care Units (Portuguese acronym: UAPS) were drawn from a total of 100 municipal UAPS equally distributed in all health regions. Users were equally chosen from the 20 units. The inclusion criteria in the sample were: adults (≥ 18 and < 60 years), literate and assisted in the different spaces of the UAPS, such as waiting rooms for medical and dental care, vaccine, dressing and reception. According to recommendations of the Item Response Theory (IRT), no sample calculation was performed, but a sufficient number of participants was used in the calibration of instrument items. No sample exclusion criteria were adopted and no one was excluded. Note that the instrument was completed by the researcher during the interview and the understanding of items was verbalized by participants and written down by the researcher.

### Analysis of results and statistics

The evidence of the validity of the NLS-BR was analyzed based on the IRT and the adoption of a two-parameter logistic model^(19)^. Thus, discriminatory power, degree of difficulty (parameters a and b, respectively) and the reliability were analyzed by the Theta (θ) coefficient. Calibration, factor loading and item information function (IIF) were considered to evaluate the permanence or exclusion of items in the NLS-BR.

The characterization data were analyzed in Microsoft Office Excel^®^ version 2007 and data related to the IRT in the IRTPRO^®^ software version 2.1. Based on Baker’s classification^(20)^, the following was adopted for “a”: very low (0.01-0.34), low (0.35-0.64), moderate (0.65-1.34), high (1.35-1.69) and very high (≥ 1.70). The level of statistical significance adopted for the tests was 5%.

Subsequently, the presence of differential item functioning (DIF) considering two age groups (age <35 years and >35 years) was analyzed. A two-dimensional model was used (items without evidence of local dependence and items with evidence). The 28 items of the NLS-BR scale were tested and 23 were calibrated via IRT in the Brazilian version.

## RESULTS

Of the 1,197 literate adults who participated in the study and responded to the Brazilian version of the NLS, the female sex predominated (75%) and the overall mean age was 36.62 (+11.68) years. Education ranged between four and 18 years of study; mean of 9.82 (+3.7) years of study. After answering the scale and the calibration of items, the final version of the NLS-BR was reached, as follows.

The NLS was translated and culturally adapted, and the final Brazilian version (NLS-BR) showed appropriate content and understanding, with evidence of validity in the target audience (Brazilian SUS users). Nutrition literacy measured by the NLS-BR was unsatisfactory (marginal and inadequate) in 36.84% of study participants, demonstrating that the understanding of nutritional information of most of them is compromised and the availability of an instrument to measure NL is relevant.

The analysis by the IRT indicated reliability of the NLS-BR for use with Brazilian SUS users. In estimating the location of individuals on the scale, the different values of Theta (θ) ranged from -2.93 to 2.30 in group 1 (≤ 35 years) and from -2.76 to 1.88 in group 2 (> 35 years), demonstrating the diversity of understanding of the studied group. Therefore, the scale would be better evaluated in a four-dimensional model and considering two groups according to age [≤ 35 years (18-35 years) and >35 years (36-60 years)].

Five items with topics related to fiber in whole grains versus processed, recommended daily amount of fiber, sugar as empty calories, organic foods versus weed control and alternate planting as weed control were excluded, originating the NLS-BR version proposed here, composed of 23 items.

Five items that did not demonstrate good discrimination in either of the two groups evaluated (≤35 years and >35 years) were excluded from the final version of the NLS-BR, in which 23 items were calibrated. When answering it, the result was classified as adequate (13-23 correct answers), marginal (7-12 correct answers) or inadequate (0-6 correct answers) literacy, the same classification adopted by Diamond (2007).

## DISCUSSION

In Brazil, there are three instruments with evidence of validity that can be used to assess NL: NVS-BR^(6)^, NLS-BR^(21)^ and the Nutrition Literacy Assessment Instrument-Nlit-Br^(13)^. The last two were developed, respectively, as a doctoral thesis and a master’s dissertation, and have not yet been published in any scientific journal. There is also a specific instrument to assess NL in diabetes, the NL among people with Diabetes (NLD) developed by Eleuterio et al.^(22)^, related to the recognition and association of words.

**Figure 1:**
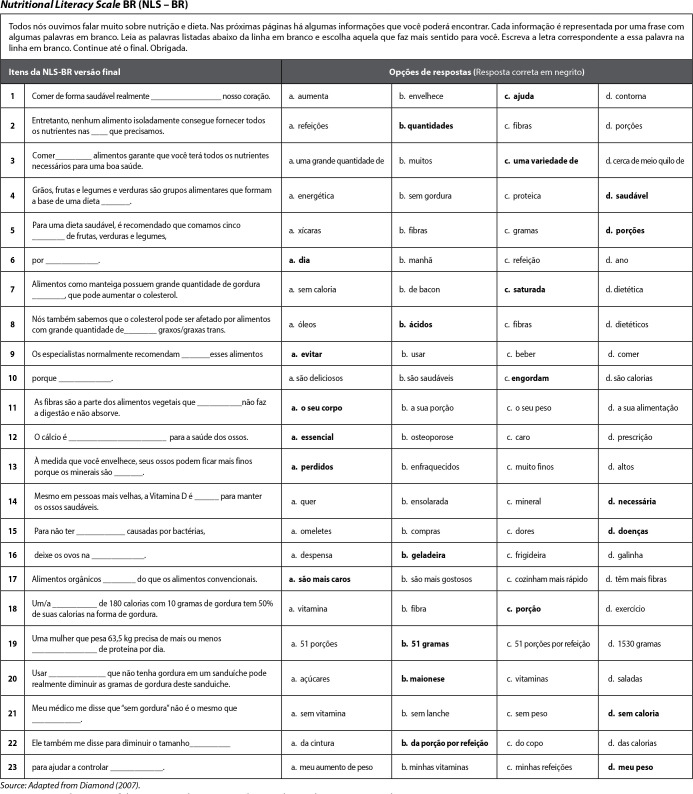
Final version of the Nutritional Literacy Scale -Brazil, Fortaleza, Ceará, Brazil, 2021

The NVS-BR^(6)^ has six items and, according to the authors of the original instrument^(5)^, the application time is three minutes and there is a strong numeracy component that may overestimate the presence of inadequate HL. A long time is needed to apply the Nlit-Br^(13)^. Although the time required for application of the NLS-BR was not timed in the present study, the study conducted by Sampaio et al.^(23)^ with a free translation of this same scale indicated that 12 minutes were necessary for the application. The NLS-BR has some particularities that make it useful for application in the Brazilian SUS, as it assesses more textual understanding than numerical ability and has a wider range of nutrition-related topics. In addition, it takes a reasonable time for completion and is not tiresome for the respondent. Its translation, adaptation and validation through IRT allowed measuring the subject’s skill in each item, analyzing parts and probabilities^(24)^ that generated the final result, making the statistical analysis more robust.

In relation to the original NLS scale, its reliability had a Cronbach’s alpha coefficient of 0.84, and a correlation of 0.61 with the Short Test of Functional Health Literacy in Adults (S-TOFHLA, an instrument widely used and accepted worldwide that measures health literacy and non-literacy in nutritional health)^(14)^. The Spanish version had a high Kuder-Richardson reliability coefficient (KR-20), a variant of Cronbach’s alpha coefficient designed for scales with binary items (KR-20 = 0.95), and the authors dichotomized the answers into correct answers and errors, suggesting robust reliability^(15)^. The Greek version of the scale had a KR of 0.94 and a Pearson’s correlation of 0.451 compared with the NVS^(16)^. Note that the Spanish scale was applied to only 134 people and the Greek version to 50 people. Therefore, the NLS-BR had a more robust statistical analysis and a larger sample, but calibrated 23 out of the 28 original items. Further studies should be conducted in other age and socioeconomic groups with a view to calibrating the five items excluded in the current version of the NLS-BR, if appropriate.

### Limitations of the Study

The population’s knowledge of NL is essential to prevent and control chronic conditions, although as already described, there are few scales to measure NL. This also makes it difficult to compare the evidence of validity of the NLS-BR with other instruments, which constitutes a limitation of this study and demonstrates the need and relevance of publishing this article with the current instrument to measure the understanding of nutritional information.

Note that the IRT was used in this article given its greater statistical robustness to assess the psychometric properties of the Brazilian version. This choice also has limitations, such as the lack of other references that allow the discussion of data from the perspective of IRT, preventing the comparison of evidence of the validity and reliability of the instrument in question.

### Contributions to the Field

However, the present study presents an expanded analysis of the scale using the IRT, which allowed greater accuracy in the analysis, including the identification and exclusion of items with low discriminatory power. In the original study by Diamond (2007), scale validation was performed only according to Cronbach’s alpha to analyze internal consistency and Pearson’s correlation to assess construct validity. After all the analyzes using the IRT, there was evidence of the content validity of the NLS-BR, even though there are still no other comparable studies.

## FINAL CONSIDERATIONS

The NLS-BR proved to be of adequate understanding, and considered as having evidence of content validity for Brazilian SUS users. The analysis with the IRT allowed statistical robustness to the NLS-BR.
